# Sigma-2 receptors as a biomarker of proliferation in solid tumours

**DOI:** 10.1054/bjoc.1999.1067

**Published:** 2000-02-21

**Authors:** K T Wheeler, L-M Wang, C A Wallen, S R Childers, J M Cline, P C Keng, R H Mach

**Affiliations:** Departments of Radiology, Physiology and Pharmacology and Pathology, Wake Forest University School of Medicine, Medical Center Boulevard, Winston-Salem, NC, 27157, USA; Cancer Center, University of Rochester Medical Center, Rochester, NY, 14642, USA

**Keywords:** Sigma-2 receptors, biomarkers, proliferative tumour cells, quiescent tumour cells, functional imaging, solid tumours

## Abstract

Over the past several years, our group has provided considerable evidence that the expression of sigma-2 (σ_2_) receptors may serve as a biomarker of tumour cell proliferation. In these in vitro studies, σ_2_receptors were expressed 8–10 times more in proliferative (P) tumour cells than in quiescent (Q) tumour cells, and the extent and kinetics of their expression were independent of a number of biological, physiological and environmental factors often found in solid tumours. Moreover, the expression of σ_2_receptors followed both the population growth kinetics when Q-cells were recruited into the P-cell compartment and the proliferative status of human breast tumour cells treated with cytostatic concentrations of tamoxifen. However, these in vitro studies may or may not be indicative of what might occur in solid tumours. In the present study, the σ_2_receptor P:Q ratio was determined for the cells from subcutaneous 66 (diploid) and 67 (aneuploid) tumours grown in female nude mice. The σ_2_receptor P:Q ratio of the 66 tumours was 10.6 compared to the σ_2_receptor P:Q ratio of 9.5 measured for the 66 tissue culture model. The σ_2_receptor P:Q ratio of the 67 tumours was 4.5 compared to the σ_2_receptor P:Q ratio of ≈ 8 measured for the 67 tissue culture model. The agreement between the solid tumour and tissue culture data indicates that: (1) the expression of σ_2_receptors may be a reliable biomarker of the proliferative status of solid tumours and (2) radioligands with both high affinity and high selectivity for σ_2_receptors may have the potential to non-invasively assess the proliferative status of human solid tumours using imaging techniques such as positron emission tomography or single-photon emission computerized tomography. © 2000 Cancer Research Campaign


*British Journal of Cancer* (2000) **82**(6), 1223–1232
© 2000 Cancer Research Campaign
Article no. bjoc.1999.1067, available online at http://www.idealibrary.com on IDEAL®

### Sigma-2 receptors as a biomarker of proliferation in solid tumours

**KT Wheeler[1], L-M Wang[1], CA Wallen[1], SR Childers[2], JM Cline[3], PC Keng[4] and RH Mach[1,2]**

Departments of [1]Radiology, [2]Physiology and Pharmacology and [3]Pathology, Wake Forest University School of Medicine, Medical Center Boulevard, Winston-Salem, NC 27157, USA; [4]Cancer Center, University of Rochester Medical Center, Rochester, NY 14642, USA

**Summary** Over the past several years, our group has provided considerable evidence that the expression of sigma-2 ($\sigma_2$) receptors may serve as a biomarker of tumour cell proliferation. In these in vitro studies, $\sigma_2$ receptors were expressed 8–10 times more in proliferative (P) tumour cells than in quiescent (Q) tumour cells, and the extent and kinetics of their expression were independent of a number of biological, physiological and environmental factors often found in solid tumours. Moreover, the expression of $\sigma_2$ receptors followed both the population growth kinetics when Q-cells were recruited into the P-cell compartment and the proliferative status of human breast tumour cells treated with cytostatic concentrations of tamoxifen. However, these in vitro studies may or may not be indicative of what might occur in solid tumours. In the present study, the $\sigma_2$ receptor P:Q ratio was determined for the cells from subcutaneous 66 (diploid) and 67 (aneuploid) tumours grown in female nude mice. The $\sigma_2$ receptor P:Q ratio of the 66 tumours was 10.6 compared to the $\sigma_2$ receptor P:Q ratio of 9.5 measured for the 66 tissue culture model. The $\sigma_2$ receptor P:Q ratio of the 67 tumours was 4.5 compared to the $\sigma_2$ receptor P:Q ratio of ≈ 8 measured for the 67 tissue culture model. The agreement between the solid tumour and tissue culture data indicates that: (1) the expression of $\sigma_2$ receptors may be a reliable biomarker of the proliferative status of solid tumours and (2) radioligands with both high affinity and high selectivity for $\sigma_2$ receptors may have the potential to non-invasively assess the proliferative status of human solid tumours using imaging techniques such as positron emission tomography or single-photon emission computerized tomography. © 2000 Cancer Research Campaign

**Keywords:** Sigma-2 receptors; biomarkers; proliferative tumour cells; quiescent tumour cells; functional imaging; solid tumours

One of the major problems in the clinical management of cancer patients is the identification of an appropriate treatment strategy for each patient. Although a number of prognostic variables that can help select a treatment strategy for a patient have been identified (e.g. tumour type, tumour size, nodal status, hypoxic fraction, proliferative status, etc.), many of these require biopsies or other invasive techniques to obtain the information (Gatenby et al, 1988; Höckel et al, 1991; Dressler, 1993; Hedley et al, 1993; Begg, 1995; Kaufman, 1996; Hayes, 1996). However, the usefulness of the information from these invasive techniques is often limited by the errors or artifacts associated with the sampling, preparation and analytical procedures.

For example, the correct choice of chemotherapeutic agents or the fractionation schedule for radiation therapy treatments frequently depends on the proliferative status of a tumour (Schabel, 1969; Thames et al, 1983; Begg et al, 1992; Corro et al, 1995, Takeda et al, 1996). It is well known that slowly proliferating tumours respond better to cell cycle non-specific agents and/or conventional radiation therapy schedules, while rapidly proliferating tumours respond better to cell cycle specific agents and/or hyperfractionated radiation therapy schedules. At the present time, the S phase fraction or the potential doubling time ($T_{pot}$) obtained flow cytometrically on biopsy specimen is used as the primary measure of a tumour's proliferative status (Begg et al, 1992; Dressler, 1993; Begg, 1995; Corro et al, 1995). Although these parameters are objective measures of a tumour's proliferative status, the biopsy specimen may not be representative of the whole tumour, and technical problems may often render unevaluable as many as 30–40% of the biopsy samples (Dressler, 1993; Hedley et al, 1993). Even when the biopsy specimens are evaluable, the results of the flow cytometric assays performed on the same sample in two or more institutions can vary enough to cause many of the patients to be treated differently (Haustermans et al, 1995; Tsang et al, 1995). Consequently, the development of a non-invasive procedure for determining the proliferative status of an entire tumour might overcome many of these problems, and permit a patient's therapy to be selected with a more uniform measure of the proliferative status as one of the prognostic variables.

A number of recent studies have reported an overexpression of sigma receptors in a variety of human and rodent tumours (Bem et al, 1991; Vilner and Bowen, 1992; Vilner et al, 1995). Sigma receptors represent a class of proteins that were originally classified as a subtype of the opiate receptors (Walker et al, 1990; Hellwell et al, 1994). Subsequent studies revealed that sigma binding sites represent a distinct class of receptors. There are two types of sigma receptors, $\sigma_1$ and $\sigma_2$. The $\sigma_1$ receptors have a molecular weight of ≈ 25 kDa, whereas the $\sigma_2$ receptors have a molecular weight of ≈ 21.5 kDa. The radioligand [$^3$H](+)-pentazocine has a high (≈ 3 nM) affinity for $\sigma_1$ receptors and a low (> 1000 nM) affinity for $\sigma_2$ receptors. The radioligand [$^3$H]1,3 di-*o*-tolyguanidine ([$^3$H]DTG) has equal affinity for both $\sigma_1$ and $\sigma_2$ receptors. Although the $\sigma_1$ receptor gene has recently been cloned from

*Received 6 May 1999*
*Revised 27 October 1999*
*Accepted 2 November 1999*

*Correspondence to:* KT Wheeler guinea pig liver, human placental choriocarcinoma, rat brain and mouse kidney (Hanner et al, 1996; Kekuda et al, 1996; Seth et al, 1997, 1998), the $\sigma_2$ receptor gene has not been cloned, probably because no high affinity selective ligand for the $\sigma_2$ receptor protein has been identified (Moebius et al, 1997). Little is known about the function of these sigma receptors (Walker et al, 1990; Vilner and Bowen, 1992; Moebius et al, 1997).

For the past several years, our group has been studying the expression of $\sigma_2$ receptors as a potential biomarker of tumour cell proliferation because every animal and human tumour cell line studied to date overexpresses $\sigma_2$ receptors when compared to the normal cell from which the tumour cell was derived (Bem et al, 1991; Vilner and Bowen, 1992; Hellwell et al, 1994; Vilner et al, 1995). Using the well-characterized in vitro mouse mammary adenocarcinoma model, lines 66 (diploid) and 67 (aneuploid), we demonstrated that the $\sigma_2$ receptor density in P-cells is 8–10 times greater than the $\sigma_2$ receptor density in Q-cells (Mach et al, 1997; Al-Nabulsi et al, 1999). In addition, we demonstrated that: (1) the kinetics for the loss of $\sigma_2$ receptors from 66 cells during the P to Q transition was identical to the kinetics for the loss of PCNA from 66 Q cells, (2) the kinetics for the increase in $\sigma_2$ receptors followed the population growth kinetics when 10-day 66 Q- or 67 Q-cells were recruited into the P-cell compartment by subculturing, (3) there was no loss of $\sigma_2$ receptors from 9L rat brain tumour cells that do not enter a Q state during the plateau phase, and (4) the reduction of $\sigma_2$ receptors from MCF-7 cells treated with cytostatic concentration of tamoxifen was quantitatively identical to the reduction in Ki-67-positive cells, AgNOR scores and the IrdU labelling index (Dong et al, 1997; Mach et al, 1997; Al-Nabulsi et al, 1999). These in vitro data suggested that $\sigma_2$ receptors may be a potential biomarker of cell proliferation in tumours, both before and after treatment.

In the study reported here, the ratio of the number of $\sigma_2$ receptors per P-cell to the number of $\sigma_2$ receptors per Q-cell was determined for both 66 and 67 tumours grown subcutaneously (s.c.) in female nude mice. It was not possible to directly measure the number of $\sigma_2$ receptors per P-cell or per Q-cell in these solid tumours for numerous technical reasons. However, the $\sigma_2$ receptor P:Q ratio could be obtained by first flow cytometrically quantitating the percentage of P- and Q-cells in one half of each tumour after labelling the tumour with bromodeoxyuridine (BrdU) every 8 h over a 48 h period, and then measuring the $\sigma_2$ receptor density per mg of tumour using our standard $\sigma_2$ binding assay on a membrane preparation from the other half of each tumour. The close agreement between the $\sigma_2$ receptor P:Q ratio determined in vitro and in situ suggests that: (1) the expression of $\sigma_2$ receptors is likely to be a reliable biomarker of the proliferative status of solid tumours and (2) radiologands that have a high affinity and a high selectivity for the $\sigma_2$ receptor have the potential to assess the proliferative status of human solid tumours using non-invasive imaging techniques such as positron emission tomography (PET) and single-photon emission computerized tomography (SPECT).

### MATERIALS AND METHODS

### Cell maintenance and tumour implantation

The 66 (diploid) and 67 (aneuploid) mouse mammary adenocarcinoma cells were grown in Weymouth's medium supplemented with 3% fetal calf serum, 6% newborn calf serum, 6% horse serum, 1% glutamine, 80.5 mg ml$^{-1}$ of streptomycin and 80.5 units ml$^{-1}$ of penicillin as previously described (Wallen et al, 1984*a*, 1984*b*). All cells were rejuvenated from frozen stock and tested for *Mycoplasma* at 3- to 6-month intervals. For the tumour implantations, exponentially growing 66 or 67 cells were trypsinized, and single cell suspensions prepared at a concentration of ≈ $10^7$ cells ml$^{-1}$ in sterile saline. Approximately $1.5 \times 10^6$ cells were injected s.c. in the inguinal region of adult (20–25 g) female nude mice to produce the tumours.

### BrdU labelling procedure

Three to four weeks after implantation, the P-cells in each tumour were identified by labelling them with BrdU (Calbiochem-Novabiochem Corp., La Jolla, CA, USA). BrdU was dissolved in 0.1 N sodium hydroxide, neutralized with 0.1 N hydrochloric acid (HCl) and diluted in sterile saline. Approximately 100 mg kg$^{-1}$ of BrdU was injected intraperitoneally (i.p.) every 8 h over a 48-h period (i.e. for at least 2 cell cycle times). At the time of labelling, the tumours ranged in size from slightly less than 0.2 g to slightly more than 2.0 g.

### Preparation of the tumours for analysis

Within 2 h of the last BrdU injection, the mice were lightly anaesthetized with Ketamine•HCl (The Butler Co., Columbus, OH, USA) and then killed by cervical dislocation. The tumour was excised sterilely, cut in half, and each half weighed. To determine the percentage of BrdU-labelled cells, the tumour was minced with fine scissors and placed in a dissociation flask with 25–30 ml of an enzyme cocktail consisting of 0.04% collagenase, 0.04% pronase (≈ 2500 PUK/100 ml) and 0.05% DNAase I in Waymouth's medium without serum. After incubating for 30–45 min at 37°C with continuous stirring, the material was filtered through an 80-mesh stainless steel filter to remove any large pieces of tissue. The filtrate was centrifuged gently at 4°C, and the pellet resuspended in Waymouth's medium. Finally, an aliquot of the cell suspension was added to an equal volume of a trypan blue solution, and the cells counted on a haematocytometer.

### Flow cytometry analysis

The single cell suspensions were gently centrifuged at 4°C, resuspended in phosphate-buffered saline (PBS) and fixed in 70% ethanol at a final concentration of $1–2 \times 10^6$ ml$^{-1}$. For the flow cytometry analysis, $1.5 \times 10^6$ cells were first incubated for 20 min at 37°C with 0.2 mg ml$^{-1}$ of pepsin in 2 N HCl–PBS, washed twice in PBS containing 0.5% fetal bovine serum (FBS), and then incubated for 45 min with a mouse anti-BrdU antibody conjugated to fluorescein isothiocyanate (Boehringer Mannheim, Indianapolis, IN, USA). The cells were then washed in 1 ml of PBS containing 0.5% FBS and 0.5% Tween-20, incubated for 30 min in RNAase (1 mg ml$^{-1}$) and stained with propidium iodide (10 μg ml$^{-1}$). All flow cytometry was performed using a Coulter Epics flow cytometer equipped with an air-cooled argon laser using an excitation wavelength of 488 nm. In each experiment, cells isolated from unlabelled 66 and 67 tumours were handled as described above in order to set the gating parameters that compensate for autofluorescence and non-specific binding of the anti-BrdU monoclonal antibody. These gating parameters were cross-checked in each experiment using unlabelled and BrdU pulse-labelled 66 and 67 tissue culture cells. The gating parameters in each experiment

© 2000 Cancer Research Campaign were set to insure that less than 2% of the unlabelled cells had a fluorescence signal equivalent to that of the weakest BrdU-labelled cells in the tumour.

### Cell survival assay

For the cell survival measurements, three dilutions of each single cell suspension were prepared and seeded into five 60-mm Petri dishes containing 5 ml of Waymouth's medium. After 10–14 days of incubation at 37°C in a humidified 5% carbon dioxide atmosphere, the medium was removed, and the colonies stained with crystal violet prior to counting.

### Morphological analysis

In one experiment, slices were cut from each half of the 66 or 67 tumours. The tumour slices were then fixed in 10% buffered formalin and embedded in paraffin. After sectioning, the tissue was stained with haematoxylin and eosin (H & E) and examined microscopically. Cytospin slides were also made from each single cell suspension, and the host cell composition of the 66 and 67 tumours was determined microscopically after staining with Wright's stain.

### $\sigma_2$ receptor density assay

A detailed description of the assay for determining the $\sigma_2$ receptor density in fmol mg$^{-1}$ of protein has been published (Mach et al, 1997). Briefly, the tumour was homogenized using a Potter-Elvejhem tissue grinder, and the membranes isolated and stored at –80°C after determining the protein content by the method of Bradford (1976). To determine the $\sigma_2$ receptor density, aliquots of the membrane preparation (30–60 μg of protein) were incubated with 4 nM [$^3$H]DTG and varying amounts of unlabelled DTG (0.1–1000 nM) in the presence of (+)-pentazocine (100 nM) to mask the $\sigma_1$ sites. After 40 min at 25°C, the assay was terminated by the addition of ice-cold 10 mM Tris–HCl (pH 8.0), and the mixture rapidly filtered using a Brandel cell harvester (Gaithersburg, MD, USA). The filters were then washed twice with ice-cold buffer and prepared for liquid scintillation counting. The saturation binding data were analysed with the Scatchard program EBDA (Biosoft, Miltown, NJ, USA) using the COLD option to calculate the $K_D$ and $B_{max}$ values. The dpm mg$^{-1}$ of each tumour was calculated using the radioactive counts obtained at $B_{max}$ and the amount of protein recovered per mg of tumour in each membrane preparation.

### P:Q ratio of $\sigma_2$ receptors in solid tumours

The number of $\sigma_2$ receptors per P-cell and per Q-cell in solid tumours cannot be determined directly by labelling the cells with BrdU and sorting the P- and Q-cells on a cell sorter for subsequent binding studies. The time required to sort an adequate number of P- and Q-cells to perform the Scatchard analysis is prohibitive. However, the P:Q ratio of the $\sigma_2$ receptors in solid tumors can be obtained by an indirect method.

If we let:

x = the fraction of Q-cells mg$^{-1}$ of tumour
1–x = the fraction of P-cells mg$^{-1}$ of tumour
k = a constant with dimensions of dpm/receptor that converts $\sigma_2$ receptors cell$^{-1}$ to dpm cell$^{-1}$ at saturation binding then:

$$\text{dpm mg}^{-1} \text{ of tumour} = \text{kx (no. of } \sigma_2 \text{ receptors per Q-cell)} + \text{k(1–x) (no. of } \sigma_2 \text{ receptors per P-cell)} \quad (1)$$

If both sides of the equation are divided by x, then:

$$\frac{\text{dpm/mg of tumour}}{\text{x}} = \text{k (no. of } \sigma_2 \text{ receptors per Q-cell)} + \text{k (no. of } \sigma_2 \text{ receptors per P-cell) ([1–x]/x)} \quad (2)$$

Equation 2 has the form Y = a + bX; the equation for a straight line. If one plots the dpm mg$^{-1}$ of tumour divided by the fraction of Q-cells mg$^{-1}$ of tumour on the *y*-axis against the fraction of P-cells mg$^{-1}$ of tumour divided the fraction of Q-cells mg$^{-1}$ of tumour on the *x*-axis (e.g. [dpm mg$^{-1}$ of tumour]/x vs [1–x]/x), a regression analysis of the data should result in a straight line with the slope equal to k (no. of $\sigma_2$ receptors per P-cell) and the intercept equal to k (no. of $\sigma_2$ receptors per Q-cell). If the slope is divided by the intercept, one obtains a ratio of the $\sigma_2$ receptors per P cell to the $\sigma_2$ receptors per Q cell for the solid tumours (Eq. 3) that can be compared to the corresponding ratio generated in tissue culture.

$$\frac{\text{slope}}{\text{intercept}} = \frac{\text{k(no. of } \sigma_2 \text{ receptors per P-cell)}}{\text{k(no. of } \sigma_2 \text{ receptors per Q-cell)}} = \text{P:Q ratio of } \sigma_2 \text{ receptors in solid tumours} \quad (3)$$

If the fraction of P-cells mg$^{-1}$ of tumour and the fraction of Q-cells mg$^{-1}$ of tumour are determined flow cytometrically on one half of the tumour after repeated injections of BrdU over a time period equal to at least 1.5–2 cell cycle times, and the dpm mg$^{-1}$ of tumour is determined from the binding of [$^3$H]DTG to the $\sigma_2$ receptors in membrane preparations from the other half of the tumour, then all of the variables in equation 2 are known for each tumour, and the $\sigma_2$ receptor P:Q ratio of each tumour can be calculated.

### Data presentation

There were six independent experiments performed during this 6-month study. Each symbol in the Figures represents one individual tumour from one of these independent experiments. Therefore, the symbols in each figure denote results from a 66 or 67 tumour in a particular independent experiment.

### RESULTS

In principle, the experimental design of this study is simple. However, a number of technical problems have the potential to seriously compromise the interpretation of the data collected in these experiments. For example, if all of the tumours in these experiments were of a similar size, the percentage of P- and Q-cells and the $\sigma_2$ receptor density in each tumour might not vary enough to generate a straight line whose slope and intercept could be determined with the accuracy necessary to have confidence in the calculated $\sigma_2$ receptor P:Q ratio. In addition, the $\sigma_2$ receptor binding studies require three replicates at each time point, so one half of the tumour must weigh ≥ 200 mg. Consequently, the tumours in the definitive experiment must have a minimum weight of ≈ 0.4 g and vary considerably in size (i.e. by a factor of ≈ 3). However, this variability in tumour size means that all technical procedures must be free of size-related artifacts if a credible $\sigma_2$ receptor P:Q ratio for these 66 and 67 tumours is going to be obtained.

*© 2000 Cancer Research Campaign* 

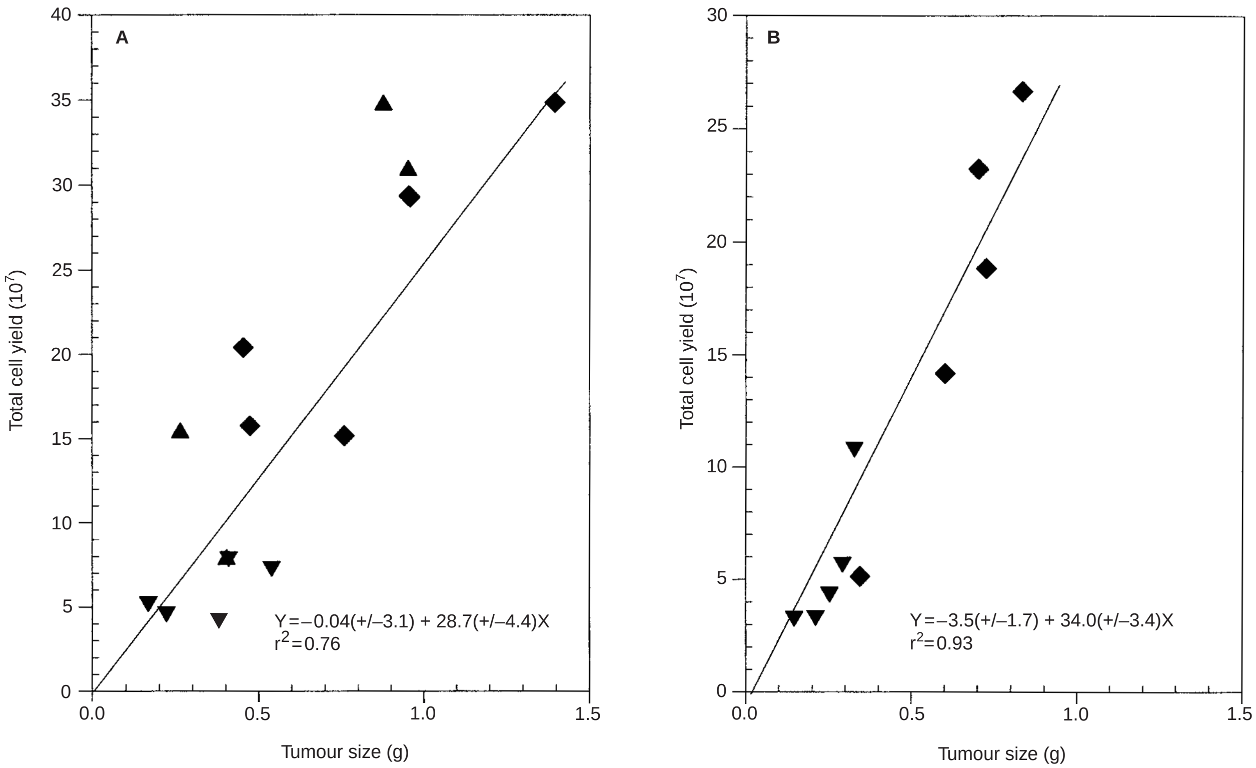

**Figure 1** Total cell yield as a function of the size of 66 (**A**) and 67 (**B**) subcutaneous tumours grown in female nude mice. The different symbols represent the results from tumours assayed in several independent experiments

### Determination of the fraction of P- and Q-cells in 66 and 67 tumours

The fraction of P-cells $mg^{-1}$ of tumour for these 66 and 67 subcutaneous tumours was estimated by injecting BrdU every 8 h for 48 h, then killing the mouse, excising the tumour, enzymatically dissociating the tumour into a single cell suspension, and flow cytometrically determining the percentage of the total cells that were labelled with BrdU. After enzymatic digestion, the total cell yield from 66 and 67 tumours was a linear function of tumour size (Figure 1). For both the 66 and 67 tumours, the slopes of the lines were similar, and the intercepts had values that were not statistically different ($P > 0.05\%$) from zero (Figure 1). Consequently, no size artifacts were apparent with our tumour cell dissociation procedure.

The absence of size-related artifacts was also supported by the morphological data obtained from the cytospin slides. Large tumour cells comprised > 70% of the cells recovered from 66 tumours and > 60% of the cells recovered from 67 tumours. Although the distribution of lymphocytes, macrophages, neutrophils and necrotic cells differed between 66 tumours and 67 tumours, there was no difference in the distribution of these host cells as a function of the tumour size (data not shown). Similarly, there was no correlation between the size of the tumour and the relative amount of necrosis present. Finally, the colony-forming efficiency for the cells isolated from 67 tumours (≈ 13%) and 66 tumours (≈ 9%) did not vary with the tumour size and was not affected by incorporation of the BrdU label. Thus, it is reasonable to conclude that a representative 66 or 67 tumour cell sample was always obtained in these experiments.

Although exponentially growing 66 and 67 tissue culture cells that were pulse-labelled with BrdU contained as much BrdU per cell as exponentially growing human tissue culture cells, the background fluorescence due to autofluorescence and non-specific binding of the monoclonal antibody to the 66 and 67 tumour cells was much higher because the commercially available antibody was produced in mice. Consequently, the signal to noise ratio was much less than that normally obtained with human tumour cells. Two gating methods were employed to estimate the percentage of BrdU-labelled 66 and 67 cells in the sample (Figure 2). The flow cytometry data were displayed either as a bivariate distribution of the BrdU content as a function of the cell's position in the cell cycle (Figure 2, row 1), or as a histogram of the BrdU content (Figure 2, row 2). Gating parameters were selected so that ≤ 2% of the unlabelled control cells (left panels) had fluorescence intensities sufficient to be considered BrdU-labelled cells. Although both methods gave identical results (Figure 2), the line method (row 2) was used to generate the data shown in the subsequent figures. The percentage of BrdU-labelled cells (P-cells) ranged from > 40% to > 70% in individual 66 and 67 tumours. There was no correlation between the percentage of P-cells and tumour size for the 66 tumours, but the number of P-cells tended to decrease with increasing tumour size for the 67 tumours (data not shown). The percentage of BrdU-labelled cells in each half of a tumour was similar (Figure 3), so it is reasonable to assume that the fraction of P-cells determined flow cytometrically in one half of a 66 or 67 tumour is representative of the fraction of P-cells in the other half of the same tumour.

*© 2000 Cancer Research Campaign*

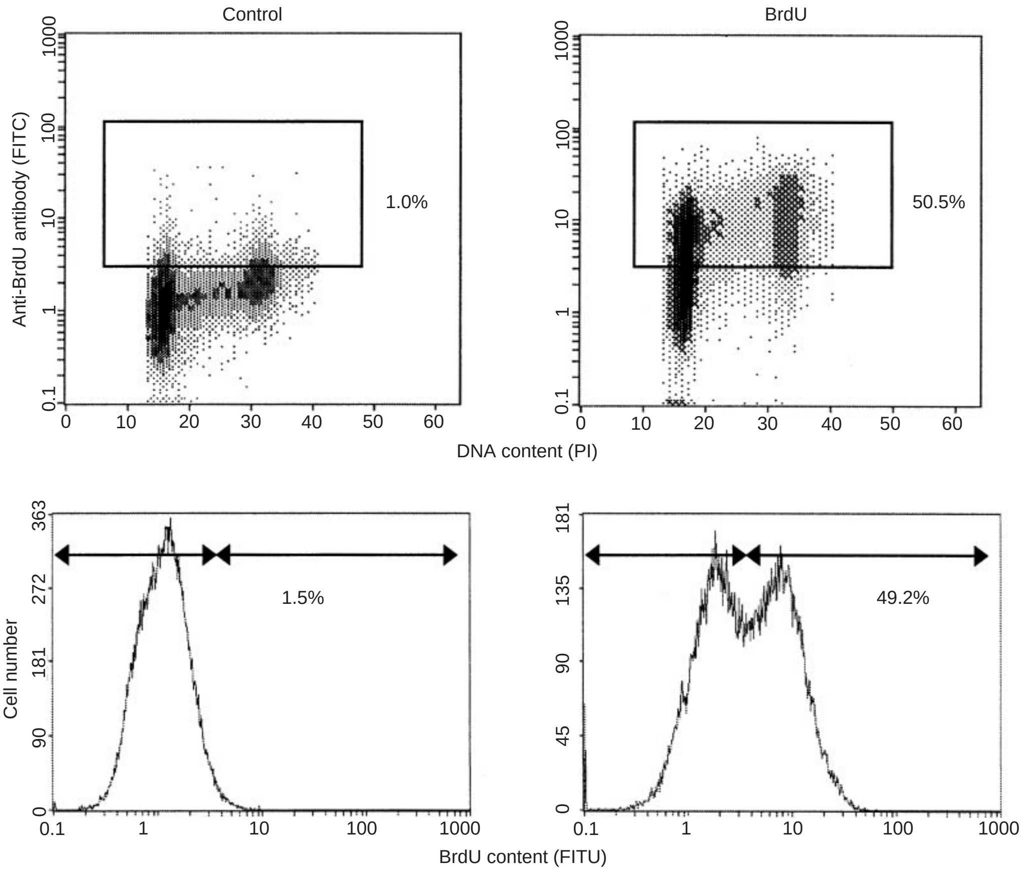

**Figure 2** Typical flow cytometry profiles for the cells separated from 66 tumours showing the two methods by which the BrdU-labelling index was determined. The control panel on the left represents the fluorescence obtained when the tumours were not labelled with BrdU. The BrdU panel on the right represents the fluorescence obtained when the tumours were labelled with BrdU (100 mg $kg^{-1}$) every 8 h for 48 h. Both the box method (row 1) and the line method (row 2) gave identical results. All of the plotted data in the subsequent figures of this report were generated by the line method (row 2)

### Determination of the $\sigma_2$ receptor density in 66 and 67 tumours

The $\sigma_2$ receptor density in 66 and 67 tumours was estimated from the binding of [$^3$H]DTG to membranes isolated from 66 and 67 tumours using (+)-pentazocine to mask the $\sigma_1$ sites. For both 66 and 67 tumours, the total membrane protein recovered was a linear function of tumour size (Figure 4). The slopes of the lines were similar for both tumours, and the intercepts had values that were not statistically different ($P$ > 0.05) from zero. The average $K_D$ value calculated from the $^3$H-DTG binding data for twelve 66 tumours (26.2 ± 10.8 nM) and ten 67 tumours (39.7 ± 16.6 nM) was slightly lower, but not statistically different ($P$ > 0.1) from the average $K_D$ values previously reported for 66 and 67 P- or Q-cells grown in tissue culture (Mach et al, 1997; Al-Nabulsi et al, 1999). In addition, the $K_D$ values for 66 and 67 tumours did not vary with tumour size (data not shown). There was no correlation between the $\sigma_2$ receptor density and tumour size for the 66 tumours, but the $\sigma_2$ receptor density decreased with increasing tumour size for the 67 tumours (Figure 5). This result is consistent with the data obtained in the BrdU-labelling study where the percentage of P-cells appeared to vary with tumour size for the 67 tumours, but not for the 66 tumours. Although the $B_{max}$ values were quite variable, ranging from ≈1000 to > 4000 fmol $mg^{-1}$ of protein for 66 tumours and from ≈750 to ≈1800 fmol $mg^{-1}$ of protein for the 67 tumours, in nine out of ten cases, the $\sigma_2$ receptor density measured in one half of a tumour was not statistically different ($P$ > 0.05) from the $\sigma_2$ receptor density measured in the other half of the same tumour (Figure 6). Thus, it is reasonable to assume that the $\sigma_2$ receptor density measured in one half of a tumour is representative of the $\sigma_2$ receptor density in the other half of the same tumour.

### Determination of the $\sigma_2$ receptor P:Q ratio in 66 and 67 tumours

When the dpm $mg^{-1}$ of tumour determined from the binding studies was divided by the fraction of Q-cells $mg^{-1}$ of tumour determined from the BrdU-labelling studies and plotted in Figure 7 against the fraction of P-cells $mg^{-1}$ of tumour divided by the fraction of Q-cells $mg^{-1}$ of tumour determined from the BrdU-labelling studies, a linear function resulted as predicted (see Materials and Methods). Dividing the slope of the line by the intercept resulted in a $\sigma_2$ receptor P:Q ratio of 10.6 for the 66 tumours and 4.5 for the 67 tumours. These values agree within a factor of 2 with the $\sigma_2$ receptor P:Q ratio of 9.5 and ≈ 8 determined from pure P and pure Q populations of 66 and 67 mouse mammary adenocarcinoma cells grown in tissue culture (Mach et al, 1997; Al-Nabulsi et al, 1999).

© 2000 Cancer Research Campaign

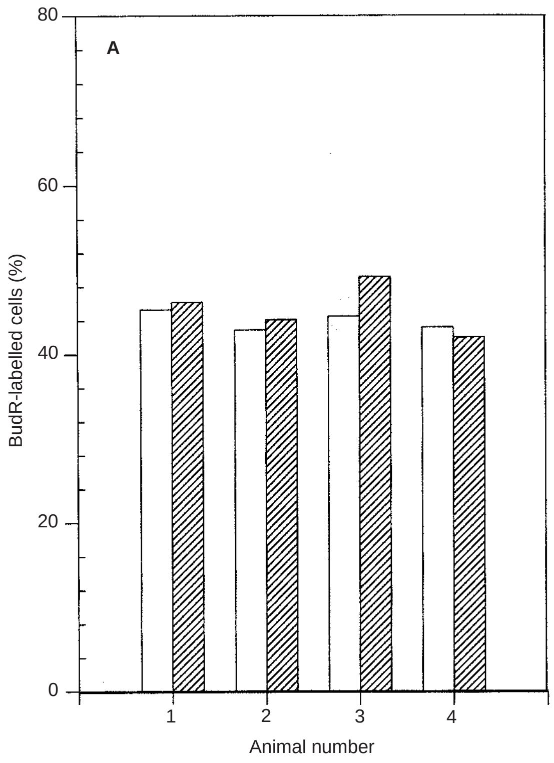

**Figure 3** Comparison of the percentage of BrdU-labelled cells determined flow cytometrically in the two halves of 66 (**A**) and 67 (**B**) subcutaneous tumours grown in female nude mice. The open bar represents one half of a tumour; the hatched bar represents the other half of the same tumour. Data from these tumours are represented in the other figures by closed upside down triangles (▼)

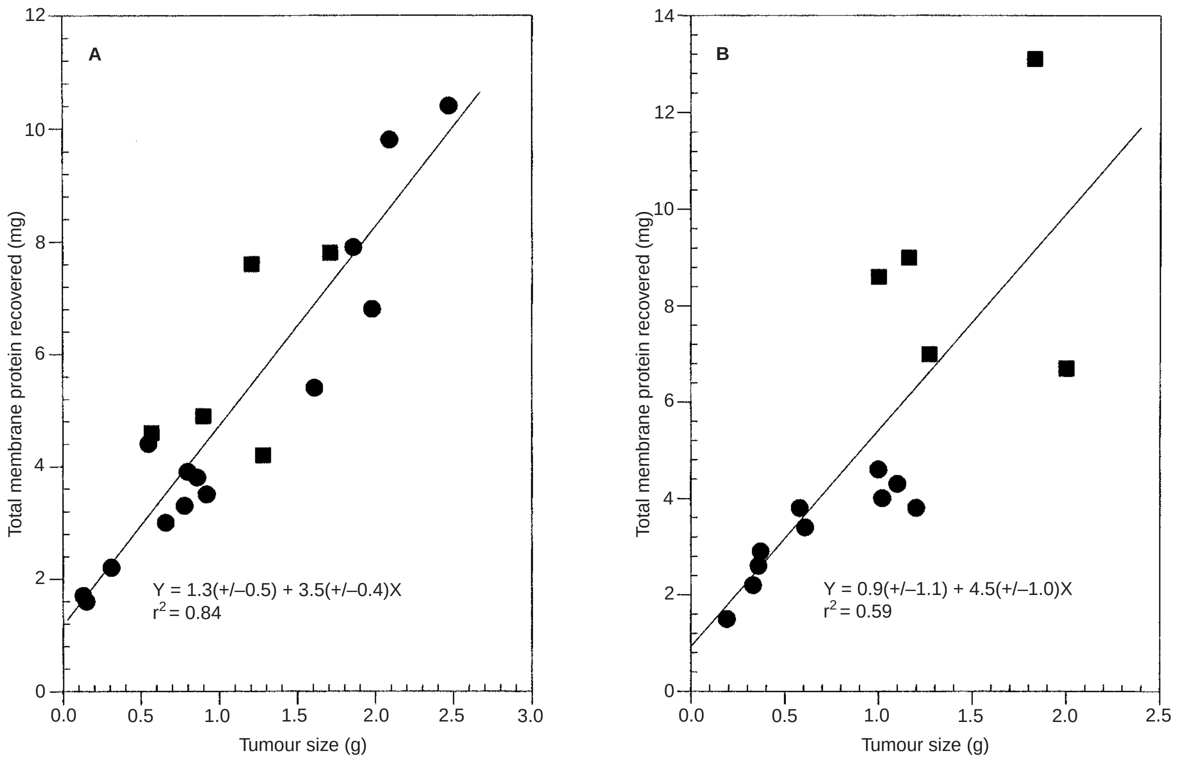

**Figure 4** Total membrane protein recovered as a function of the size of 66 (**A**) and 67 (**B**) subcutaneous tumours grown in female nude mice. The different symbols represent the results from tumours assayed in two independent experiments

 *© 2000 Cancer Research Campaign*

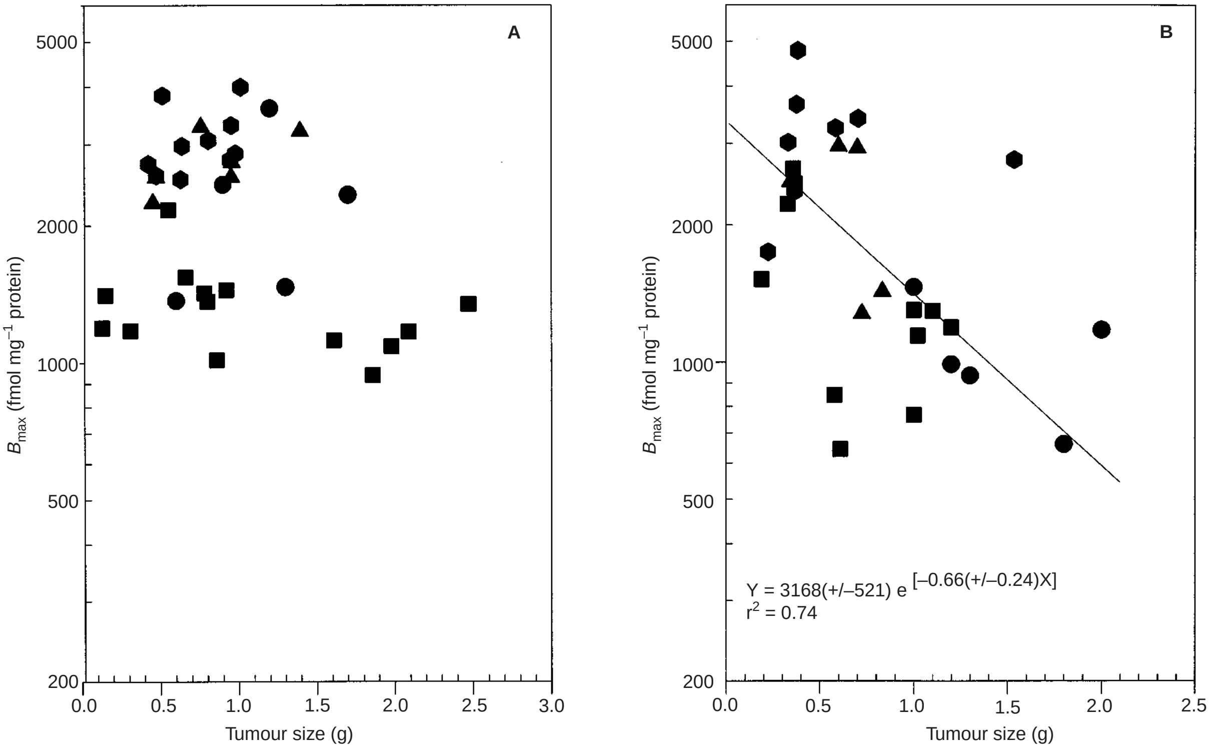

**Figure 5** $\sigma_2$ receptor density ($B_{max}$) as a function of the size of 66 (**A**) and 67 (**B**) subcutaneous tumours grown in female nude mice. The different symbols represent the results from tumours assayed in four independent experiments

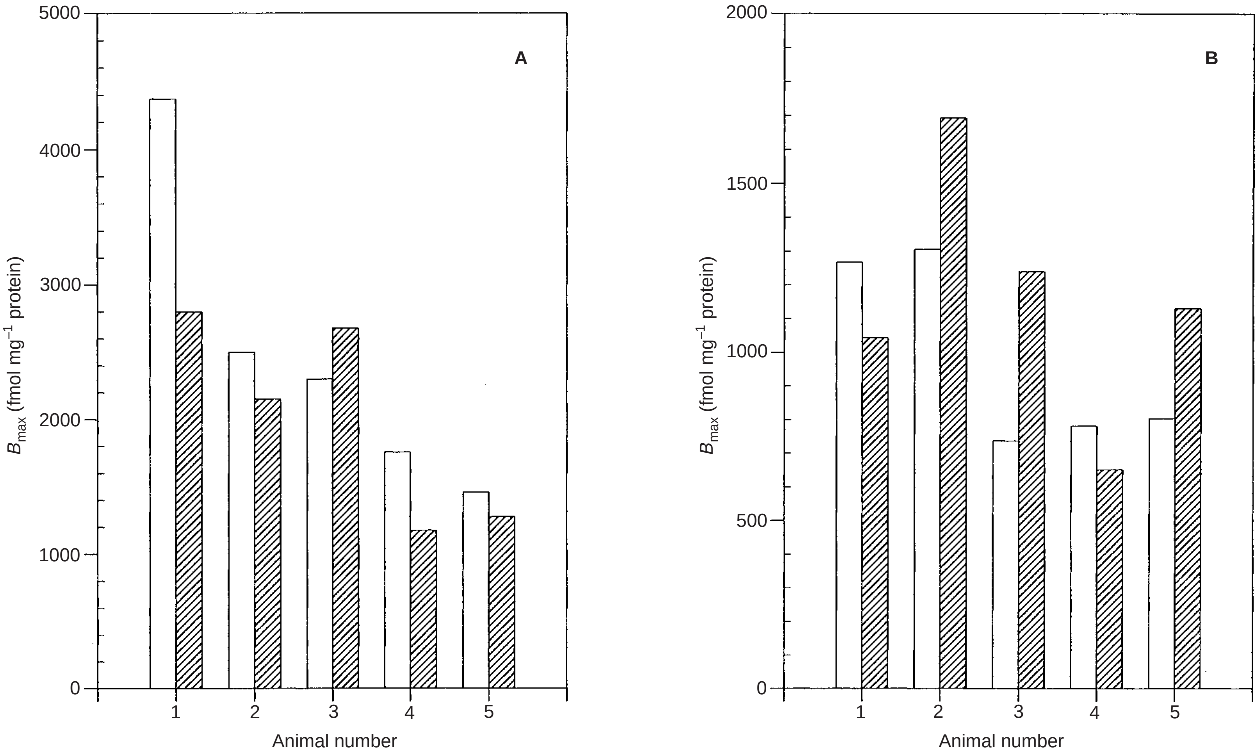

**Figure 6** Comparison of the $\sigma_2$ receptor density ($B_{max}$) determined in the two halves of 66 (**A**) and 67 (**B**) subcutaneous tumours grown in female nude mice. The open and hatched bars are data from each half of a tumour as described in the legend to Figure 3. Data from these tumours are represented in the other figures by closed circles (●)

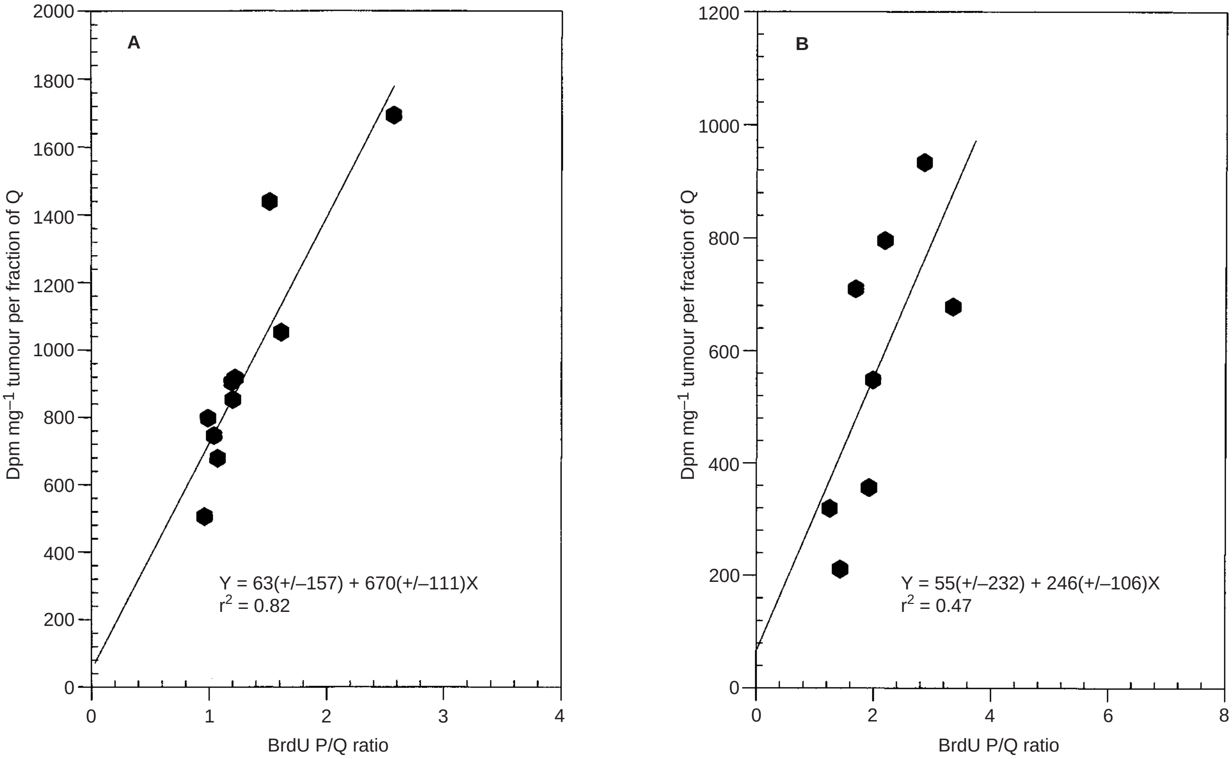

**Figure 7** Determination of the ratio of the number of $\sigma_2$ receptors per P-cell to the number of $\sigma_2$ receptors per Q-cell in subcutaneous 66 (**A**) and 67 (**B**) tumours grown in female nude mice. The equation was generated by a least squares linear regression analysis of the data, and the $\sigma_2$ receptor P:Q ratio of 10.6 for 66 tumours and 4.5 for 67 tumours was calculated by dividing the slope of the corresponding equation by the intercept (see Materials and Methods for details)

### DISCUSSION

### Estimation of the P-cell compartment

In general, tumour cells in animals must be labelled with a DNA precursor for 1.5–2 cell cycle times to ensure that all of the cycling cells (P-cells) have been labelled. For example, although 9L rat brain tumour cells have an average cell cycle and population doubling time of ≈ 20 h, time-lapse photography studies have shown that, in an exponentially growing population, individual 9L cells have cell cycle times as short as 13 h and as long as 35 h (Ehmann and Wheeler, 1979). Despite this variability in cell cycle times, all of these 9L cells were capable of dividing enough times (> 6) to form a colony. A similar heterogeneity in cell cycle times has also been described for other tumour cell lines (Ehmann et al, 1974). In our lab, 66 cells have a population doubling time in tissue culture of 16.0 ± 1.0 h; 67 cells have a population doubling time in tissue culture of 15.8 ± 0.3 h (Mach et al, 1997; Al-Nabulsi et al, 1999). In this study, the 66 tumours had a volume doubling time of about 24 h, and the 67 tumours had a volume doubling time of about 30 h. Nothing else is known about the in situ cell kinetics of these 66 and 67 tumours at this time. In selecting the 48 h BrdU-labelling period, it was assumed that the average in situ cell cycle time for these 66 and 67 tumours would be at least as long as that measured in tissue culture, and probably not as long as the tumour volume doubling time. If the in situ cell cycle time for these tumour cells were as short as that measured in tissue culture, the percentage of BrdU-labelled cells measured in this study would slightly overestimate the fraction of P-cells $mg^{-1}$ of tumour. However, slightly overestimating the P-cell compartment is not expected to have a large effect on the $\sigma_2$ receptor P:Q ratio determined from the data in Figure 7.

### Estimation of the $\sigma_2$ receptor density

The presence of host cells (lymphocyte, macrophages, neutrophils, etc.) in the tumour also has the potential to complicate the interpretation of these data. Most tumours are comprised of 30–60% host cells that are predominantly non-proliferative or quiescent (Siemann et al, 1984). The $\sigma_2$ receptor density of these terminally differentiated host cells is unknown, but it is likely to be closer to that of Q tumour cells than P tumour cells. Finally, the fraction of the protein in the membrane preparation that comes from these host cells is also unknown. Fortunately, most of the cells on the cytospin slides prepared from nine 66 and nine 67 tumours were clearly identified as large tumour cells (62 ± 12% and 71 ± 11% respectively). Another 20± 8% of the cells from the 67 tumours were either small tumour cells or lymphocytes that could not be distinguished from each other. The small tumour cell/lymphocyte component of the 66 tumours was 12 ± 5%. If it is assumed that half of these small cells are tumour cells, > 80% of the cells in 67 tumours and ≈ 70% of the cells in 66 tumours are tumour cells. Given that, of the host cells, only the macrophages have a size similar to the large tumour cells, and macrophages comprise only 8± 5% of the cells from 67 tumours and 10 ± 3% of the cells from 66 tumours, it is reasonable to suggest that > 90% of the protein in the membrane preparations from 67 tumours and > 80% of the protein in the membrane preparations from 66 tumours came from tumour cells, not host cells. Although the presence of host cells in these tumours will tend to bias the $\sigma_2$ receptor binding results by

 © 2000 Cancer Research Campaign overestimating the Q-cell component, the close agreement between the $\sigma_2$ receptor P:Q ratios measured for these 66 and 67 subcutaneous tumours and the 66 and 67 cells in tissue culture indicates that the bias was minimal. This result is expected if > 80% of the membrane protein for the binding studies came from 66 or 67 tumour cells. It should also be noted that a slight overestimation of the P-cell component in the BrdU-labelling studies might have compensated for a slight overestimation of the Q-cell component in the binding studies, when the in situ $\sigma_2$ receptor P:Q ratio was calculated from the data in Figure 7.

### Tumour size as a determinant of the P:Q ratio

In general, the fraction of Q cells in a tumour is anticipated to increase as the tumour size increases and the tumour volume doubling time increases. Both the BrdU-labelling data and the $\sigma_2$ receptor binding data (Figure 5) indicate that this generalization is true for 67 tumours, but not for 66 tumours. Although the reason for this difference between the tumours is not known, having an in situ tumour model, where $B_{max}$ and the fraction of P- and Q-cells is relatively constant as a function of tumour size, can be quite valuable when performing preclinical biodistribution studies of candidate $\sigma_2$-selective radioligands. If 66 tumours are used, tumour size contributes minimally to the variability of the biodistribution data; thereby making these experiments more efficient, more convenient and less costly.

### Comparison of the $\sigma_2$ receptor P:Q ratio in vivo and in vitro

Usually, a statistical analysis of the data would be sufficient to conclude that the $\sigma_2$ receptor P:Q ratio in 66 and 67 solid tumours was identical to the $\sigma_2$ receptor P:Q ratio obtained with pure P and pure Q populations of the same cells in tissue culture. However, the parameters used to construct the $\sigma_2$ receptor P:Q ratio have very large errors associated with them because both the experimental design and the techniques used in the experiments are complicated (Figure 7) (Mach et al, 1997; Al-Nabulsi et al, 1999). For example, the day to day variability in the $^3$H-DTG binding data required that the 66 or 67 P- and Q-cells be harvested from tissue culture and analysed as matched pairs in order to obtain a reliable estimate of the $\sigma_2$ receptor P:Q ratio (Mach et al, 1997; Al-Nabulsi et al, 1999). When the errors on the individual parameters were used to calculate the errors on the $\sigma_2$ receptor P:Q ratio, the 95% confidence interval was as large as the P:Q ratio for the tissue culture data, and more than 5 times the P:Q ratio for the solid tumour data. Consequently, a statistical analysis of the data would not allow rejection of the null hypothesis that the in vivo and in vitro $\sigma_2$ receptor P:Q ratios are equal, unless the in vivo value is several times larger or smaller than the in vitro value.

Being cognizant of this potential problem, the samples for the in vivo experiments were collected and blinded by one group of investigators, and the critical components of the data were then measured by two other groups of investigators at two different institutions. After uncoding, the data was recombined to create the points shown in Figure 7. Given that: (1) the limited data set for each of the tumours could have exhibited a negative correlation, a positive correlation, or no correlation and (2) the slope:intercept ratio of any positive correlation (i.e. the $\sigma_2$ receptor P:Q ratio) could have varied from very large negative values to very large positive values, it is remarkable that the in vivo and in vitro estimates of the $\sigma_2$ receptor P:Q ratio agree within a factor of 2. Thus, it is not the statistical analysis of the data, but rather the close agreement between the in vivo and in vitro $\sigma_2$ receptor P:Q ratios which constitutes the most convincing argument for the claim that the in vivo and in vitro $\sigma_2$ receptor P:Q ratios are actually equal.

### Implications for determining the proliferative status of solid tumours by imaging

In recent years, several potential noninvasive imaging approaches to assessing the proliferative status of solid tumours have been investigated. In general, these approaches have involved the use of radioligands that target a variety of metabolic processes that are likely to vary with the proliferative status of a tumour. Examples include: (1) [$^{18}$F]FDG which measures glucose utilization; (2) [$^{11}$C]methionine ([$^{11}$C]MET) which measures the rate of protein synthesis, and [$^{11}$C]thymidine ([$^{11}$C]TdR) which measures the rate of DNA synthesis (Minn et al, 1988; Martiat et al, 1988; Minn and Soini, 1989; Leskinen-Kallio et al, 1991; Haberkorn et al, 1991; Okada et al, 1992; Miyazawa et al, 1993). However, the correlation coefficients for these metabolic biomarkers of cell proliferation with other known biomarkers of cell proliferation have ranged from 0.6 to 0.8; values that are not sufficiently high to allow a patient's treatment to be individualized based on the results of these metabolic imaging procedures.

Over the past several years, $\sigma_2$ receptors have been found on all of the animal and human tumour cell lines that have been studied (Bem et al, 1991; Vilner and Bowen, 1992; Vilner et al, 1995). In general, the number of $\sigma_2$ receptors per tumour cell is equal to or exceeds the number of $\sigma_1$ receptors per tumour cell. Using several tissue culture models, our lab has demonstrated that $\sigma_2$ receptors appear to be a biomarker of tumour cell proliferation (Mach et al, 1997; Al-Nabulsi et al, 1999). However, if the proliferative status of solid tumours is to be determined using radioligands that bind to $\sigma_2$ receptors and imaging techniques such as PET: (1) the $\sigma_2$ receptor P:Q ratio in situ should at least exceed three (Amano et al, 1998), (2) radioligands with a high selectivity and high affinity for $\sigma_2$ receptors must be available (Moebius et al, 1997) and (3) endogeneous ligands must not interfere significantly with the binding of the radiopharmaceutical in tumours and normal tissues (Liu et al, 1992). The data in Figure 7 not only demonstrate that the in situ $\sigma_2$ receptor P:Q ratio exceeds three for both 66 and 67 mouse mammary tumours, but that the in situ $\sigma_2$ receptor P:Q ratio is also in reasonable agreement with the 8–10 obtained in studies using the corresponding tissue culture models. Secondly, our group has recently synthesized a ligand that, in preliminary binding studies, has both a high selectivity (> 30-fold) and a high affinity ($\approx$ 3 nM) for $\sigma_2$ receptors. Finally, we have preliminary data using a non-selective $\sigma_2$ radioligand and a $\sigma_1$-selective blocking agent to label 66 tumours in female nude mice, which indicate that tumour:blood, tumour:muscle and tumour:lung ratios of at least 15:1 should be achievable with a $\sigma_2$-selective radioligand having an affinity of $\approx$ 5 nM (unpublished data). Consequently, combining the data in Figure 7 which indicate that the expression of $\sigma_2$ receptors may be a reliable biomarker of proliferation in solid tumours, with our preliminary data on the properties and biodistribution of radioligands that bind selectively to $\sigma_2$ receptors, makes it likely that the proliferative status of solid tumors can be non-invasively assessed using PET and/or SPECT imaging techniques in the near future.

*© 2000 Cancer Research Campaign*

### ACKNOWLEDGEMENTS

All tissue culture medium and *Mycoplasma* testing were provided by the Tissue Culture Core Laboratory of the Comprehensive Cancer Center of Wake Forest University (CCCWFU) which is supported in part by NIH grant CA-12197. The statistical analyses were performed with the help of Dr D Case of the Biostatistical Unit of the CCCWFU. The flow cytometry analysis was performed in the University of Rochester Cancer Center Flow Cytometry Facility. We thank K Sten for technical support during the pilot phase of this project, and B Tyler for helping to prepare the manuscript.

### REFERENCES

Al-Nabulsi I, Mach RH, Wang L-M, Wallen CA, Keng PC, Sten K, Childers SR and Wheeler KT (1999) Effect of ploidy, recruitment, environmental factors, and tamoxifen on the expression of sigma-2 receptors in proliferating and quiescent tumour cells. *Br J Cancer* **81**: 925–933

Amano S, Inoue T, Tomiyoshi K, Ando T and Endo K (1998) In vivo comparison of PET and SPECT radiopharmaceuticals in detecting breast cancer. *J Nucl Med* **39**: 1424–1427

Begg AC (1995) The clinical status of $T_{pot}$ as a predictor? Or why no tempest in the $T_{pot}$! *Int J Radiat Oncol Biol Phys* **32**: 1539–1541

Begg AC, Hofland I, Van Glabbeke M and Horiot JC (1992) Predictive value of potential doubling time for radiotherapy of head and neck tumor patients. Results from the EORTC cooperative trial 22851. *Semin Rad Oncol* **1**: 22–25

Bem WT, Thomas GE, Mamone JY, Homan SM, Levy BK, Johnson FE and Coscia CJ (1991) Overexpression of σ receptors in nonneural human tumors. *Cancer Res* **51**: 6558–6562

Bradford MM (1976) A rapid and sensitive method for quantitation of microgram quantities of protein utilizing the principle of protein-dye binding. *Anal Biochem* **72**: 248–254

Corro R, Giaretti W, Sanguinet G, Geido E, Orechia R, Guenzi M, Margarino G, Bacigalupo A, Garaventa G, Barbieri M and Vitale V (1995) In vivo cell kinetics in head and neck squamous cell carcinomas predicts local control and helps guide radiotherapy regimen. *J Clin Oncol* **13**: 1843–1850

Dong H, Bertler C, Schneider E and Ritter MA (1997) Assessment of cell proliferation by AgNOR scores and Ki-67 labeling indices and a comparison with potential doubling times. *Cytometry* **28**: 280–288

Dressler LG (1993) DNA flow cytometry measurements and their clinical relevance in node-negative breast cancer patients. *Recent Results Cancer Res* **127**: 61–69

Ehmann UK and Wheeler KT (1979) Cinemicrographic determination of progression and division abnormalities after treatment with 1,3 bis(2-chloroethyl)-1-nitrosourea. *Eur J Cancer* **15**: 461–473

Ehmann UK, Nagasawa H, Petersen DF and Lett JT (1974) Symptoms of X-ray damage to radiosensitive mouse leukemic cells: asynchronous populations. *Radiat Res* **60**: 453–472

Gatenby RA, Kessler HB, Rosenblum JS, Coia LR, Moldofsky PJ, Hartz WH and Broder GJ (1988) Oxygen distribution in squamous cell carcinoma metastases and its relationship to outcome of radiation therapy. *Int J Radiat Oncol Biol Phys* **14**: 831–838

Haberkorn U, Strauss JG, Reisser CH, Haag D, Dimitrakopoulou A, Ziegler S, Oberdorfer F, Rudat VA and Van Kaick G (1991) Glucose uptake, perfusion and cell proliferation in head and neck tumors: relation of positron emission tomography to flow cytometry. *J Nucl Med* **32**: 1548–1555

Hanner M, Moebius FF, Flandorfer A, Knaus H-G, Striessnig J, Kempner E and Glossman H (1996) Purification, molecular cloning and expression of the mammalian $sigma_1$ binding site. *Proc Natl Acad Sci USA* **93**: 8072–8077

Haustermans K, Hofland I, Pottie G, Ramakers M and Begg AC (1995) Can measurements of potential doubling time ($T_{pot}$) be compared between laboratories? A quality control study. *Cytometry* **19**: 154–163

Hayes DF (1996) Serum (circulating) tumor markers for breast cancer. *Recent Results Cancer Res* **140**: 101–113

Hedley DW, Clark GM, Cornelisse CJ, Killander D, Kute T and Merkel D (1993) Consensus review of the clinical utility of DNA cytometry in carcinoma of the breast. *Cytometry* **14**: 482–485

Hellwell SB, Bruce A, Feinstein G, Orringer J, Williams W and Bowen WD (1994) Rat liver and kidney contain high densities of $\sigma_1$ and $\sigma_2$ receptors: characterization by ligand binding and photoaffinity labeling. *Eur J Pharmacol Mol Pharmacol Sec* **268**: 9–18

Höchel M, Schlenger K, Knoop C and Vaupel P (1991) Oxygenation of carcinomas of the uterine cervix: Evaluation of computerized $O_2$ tension measurements. *Cancer Res* **51**: 6098–6102

Kaufman M (1996) Review of known prognostic variables. *Recent Results Cancer Res* **140**: 77–87

Kekuda R, Prasad P, Fei Y-J, Leibach FH and Ganapathy V (1996) Cloning and functional expression of the human type one sigma receptor (hSigma R1). *Biochem Biophys Res Commun* **223**: 553–558

Leskinen-Kallio S, Nagren K, Lehikoinen P, Ruotsalainen U and Joensuu H (1991) Uptake of $^{11}$C-methionine in breast cancer studied by PET. An association with the size of the S-phase fraction. *Br J Cancer* **64**: 1121–1124

Liu A, Dence CS, Welch MJ and Katzenellenbogen JA (1992) Fluorine-18-labeled androgens: radiochemical synthesis and tissue distribution studies on six fluorine-substituted androgens, potential imaging agents for prostatic cancer. *J Nucl Med* **33**: 724–734

Mach RH, Smith CR, Al-Nabulsi I, Whirrett BA, Childers SR and Wheeler KT (1997) $\sigma_2$ receptors as potential biomarkers of proliferation in breast cancer. *Cancer Res* **57**: 156–161

Martiat PH, Ferrant A, Labar D, Cogneau M, Bol A, Michael C, Michaux JL and Sokal G (1988) In vivo measurement of carbon-11 thymidine uptake in non-Hodgkins lymphoma using positron emission tomography. *J Nucl Med* **29**: 1633–1637

Minn H and Soini I (1989) [$^{18}$F]fluorodeoxyglucose scintigraphy in diagnosis and follow-up of treatment in advanced breast cancer. *Eur J Nucl Med* **15**: 61–66

Minn J, Joensuu H, Ahonen A and Klemi P (1988) Fluorodeoxyglucose imaging: a method to assess proliferative activity of human cancer in vivo. *Cancer* **61**: 1776–1781

Miyazawa H, Arai T, Iio M and Hara T (1993) PET imaging of non-small cell lung carcinoma with carbon-11-methionine: relationship between radioactivity uptake and flow-cytometric parameters. *J Nucl Med* **43**: 1886–1891

Moebius FF, Stiessnig J and Glossman H (1997) The mysteries of the sigma receptors: new family members reveal a role in cholesterol synthesis. *TIPS* **18**: 67–70

Okada J, Yoshikawa K, Itami M, Imaseki K, Uno K, Itami J, Kuyama J, Mikata A and Arimizu N (1992) Positron emission tomography using fluorine-18-fluorodeoxyglucose in malignant lymphoma: a comparison with proliferative activity. *J Nucl Med* **33**: 325–329

Schabel FM (1969) The use of tumor growth kinetics in planning "curative" chemotherapy of advanced solid tumors. *Cancer Res* **29**: 2384–2389

Seth P, Leibach FH and Ganapathy V (1997) Cloning and structural analysis of the cDNA and the gene encoding the murine type 1 sigma receptor. *Biochem Biophys Res Commun* **241**: 535–540

Seth P, Fei Y-J, Li HW, Huang W, Leibach FH and Ganapathy V (1998) Cloning and functional characterization of a σ receptor from rat brain. *J Neurochem* **70**: 922–931

Siemann DW, Lord EM, Keng PC and Wheeler KT (1984) Cell subpopulations dispersed from solid tumors and separated by centrifugal elutriation. *Br J Cancer* **44**: 100–108

Takeda N, Diksic M and Yamamoto YL (1996) The sequencial changes in DNA synthesis, glucose utilization, protein synthesis, and peripheral benzodiazepine receptor density in C6 brain tumors after chemotherapy to predict the response of tumors to chemotherapy. *Cancer* **77**: 1167–1179

Thames HD, Peters LJ, Withers HR and Fletcher GH (1983) Accelerated fractionation vs hyperfractionation: rationales for several treatments per day. *Int J Radiat Oncol Biol Phys* **9**: 127–138

Tsang RW, Fyles AW, Kirkbride P, Levin W, Manchul LA, Milosevic MF, Rawlings GA, Banerjee D, Pinitilie M and Wilson GD (1995) Proliferation measurements with flow cytometry $T_{pot}$ in cancer of the uterine cervix: correlation between two laboratories and preliminary clinical results. *Int J Radiat Oncol Biol Phys* **32**: 1319–1329

Vilner BJ and Bowen WD (1992) Characterization of sigma-like binding properties of NB41A3, S-20Y, and N1E-115 neuroblastoma, C6 glioma, and NG108–15 neuroblastoma-glioma hybrid cells. Further evidence for sigma receptors. In: *Multiple Sigma and PCP Receptor Ligands: Mechanisms for Neuromodulation and Neuroprotection*, Kamenka JM, Domino EF (eds) pp 341–353. NPP Books: Ann Arbor

Vilner BJ, John CS and Bowen WD (1995) Sigma-1 and sigma-2 receptors are expressed in a wide variety of human and rodent tumor cell lines. *Cancer Res* **55**: 408–413

Walker JM, Bowen WD, Walker FO, Matsumoto RE, De Costa B and Rice KR (1990) Sigma receptors: biology and function. *Pharmacol Rev* **42**: 355–402

Wallen CA, Higashikubo R and Dethlefsen LA (1984*a*) Murine mammary tumor cells *in vitro*. I. The development of a quiescent state. *Cell Tissue Kinet* **17**: 65–77

Wallen CA, Higashikubo R and Dethlefsen LA (1984*b*) Murine mammary tumor cells *in vitro*. II. Recruitment of quiescent cells. *Cell Tissue Kinet* **17**: 79–89

 *© 2000 Cancer Research Campaign*
